# Small Sample Building Energy Consumption Prediction Using Contrastive Transformer Networks

**DOI:** 10.3390/s23229270

**Published:** 2023-11-19

**Authors:** Wenxian Ji, Zeyu Cao, Xiaorun Li

**Affiliations:** 1 College of Electrical Engineering, Zhejiang University, 866 Yuhangtang Road, Hangzhou 310058, China; 11910094@zju.edu.cn; 2 School of Spatial Planning and Design, Hangzhou City University, 51 Huzhou Street, Hangzhou 310015, China; caozy@hzcu.edu.cn

**Keywords:** small sample learning, contrastive learning, energy consumption prediction

## Abstract

Predicting energy consumption in large exposition centers presents a significant challenge, primarily due to the limited datasets and fluctuating electricity usage patterns. This study introduces a cutting-edge algorithm, the contrastive transformer network (CTN), to address these issues. By leveraging self-supervised learning, the CTN employs contrastive learning techniques across both temporal and contextual dimensions. Its transformer-based architecture, tailored for efficient feature extraction, allows the CTN to excel in predicting energy consumption in expansive structures, especially when data samples are scarce. Rigorous experiments on a proprietary dataset underscore the potency of the CTN in this domain.

## 1. Introduction

In recent years, numerous countries have mandated “low-carbon” and “energy-saving” criteria for the construction and operation of buildings as a commitment to environmental protection [1,2]. The emphasis on curtailing the energy demands of building operations has positioned the accurate prediction of electricity consumption at the forefront of research for many engineers and scholars. Precise forecasting of future electricity consumption during building operation and maintenance can inform judicious electricity procurement strategies and guide equipment selection. This not only considerably trims the economic costs associated with operation and maintenance but also paves the way for the realization of low-carbon, energy-efficient buildings. By optimizing energy utilization, buildings can diminish their excessive consumption, thereby reducing their carbon footprint. This fosters enhanced load shifting, seamless integration of renewable energy, and the actualization of energy efficiency measures.

At present, numerous studies related to building energy consumption prediction have been presented. Divina et al. [3] employed a range of machine learning techniques to predict energy consumption in smart buildings. Their research provided a comparative analysis utilizing data from thirteen buildings on a university campus, shedding light on the performance of various machine learning approaches. Sehovac et al. [4] employed recurrent neural networks (RNNs) and sequence-to-sequence (S2S) deep learning models for energy load predictions. Their results underscored the models’ capability to efficiently process time-series data, resulting in precise short-term forecasts. Haq et al. [5] amalgamated convolutional long short-term memory (ConvLSTM) with bidirectional long short-term memory (BiLSTM) to predict energy consumption in both residential and commercial sectors. Their combined model exhibited enhanced accuracy and stability, particularly when handling multi-modal sensor data. Khan et al. [6] put forth an ensemble technique, integrating long short-term memory (LSTM) with a Kalman filter (KF), targeting short-term energy consumption predictions in multifamily residential buildings. Their approach leveraged LSTM’s adeptness at sequence modeling with the KF’s noise filtering capabilities, producing predictions that are both consistent and applicable in real-world scenarios. Olu-Ajayi et al. [7] delved into the appropriateness of different machine learning techniques for projecting potential residential building energy consumption during the early design phase, aiming to mitigate the creation of energy-inefficient structures. Wenninger et al. [8] introduced the innovative QLattice algorithm, applying it to a dataset encompassing over 25,000 German residential buildings. Their goal was to predict annual building energy performance while emphasizing the balance between predictive accuracy and the potential of explainable artificial intelligence.

Although numerous methods have been proposed for predicting building energy consumption [9], a majority of these forecasting approaches necessitate vast amounts of training data to ensure robust predictive accuracy. In situations with consistent electricity usage patterns, acquiring data is relatively straightforward. For instance, utilizing electricity consumption data from multiple residents in an apartment complex to estimate the energy usage of other inhabitants, or harnessing data from several office buildings to forecast the energy demands of similarly purposed offices. However, unique structures, such as exhibition centers designated for trade shows, often exhibit electricity consumption patterns that deviate from residential and conventional office edifices. Acquiring data from analogous buildings to aid in prediction becomes challenging, culminating in a scarcity of training data for forecasting electricity consumption in these sizable establishments. Considering the high energy demands of such structures, precise prognostications of future energy usage could yield significant financial savings. Consequently, addressing the energy consumption forecasting challenges for these edifices is of paramount research significance.

Given the contemporary research landscape, deep learning algorithms manifest superior performance in contexts abundant in data [10]. Conversely, in situations marked by data paucity, traditional machine learning techniques tend to excel [11]. This differential efficacy can be attributed to the inherent data-centric nature of deep learning algorithms, making them profoundly reliant on the volume of the training data. Traditional machine learning approaches, in juxtaposition, discern pivotal samples from the training dataset, utilizing them as foundational elements for crafting a predictive model. Nonetheless, such strategies are often vulnerable to anomalies in the training samples. These anomalies or outliers can inadvertently infuse biases into the predictive framework. A pertinent example of this sensitivity is evident in models like the support vector machine (SVM) [12]. To circumvent data-induced biases whilst optimally harnessing the information encapsulated within the dataset, self-supervised learning emerges as a potent and forward-looking deep learning paradigm.

Self-supervised learning autonomously generates labels by orchestrating a pseudo-task, typically entailing the prediction of specific segments of data, be it regions within an image or words in a textual context [13]. This methodology obviates the necessity for labor-intensive manual labeling. In the aegis of these pseudo-tasks, adeptly crafted network architectures assimilate data features through contrasting input samples. This dynamic culminates in network weights that are optimally tuned for subsequent task training. Pertinent research has unequivocally showcased that integrating self-supervised learning can effectively diminish the reliance of deep learning architectures on voluminous training data, thereby broadening the purview of deep learning techniques [14].

Momentum Contrast (MoCo), pioneered by He et al. [15], emerged as a response to the challenges of extracting meaningful representations from unlabeled datasets. MoCo utilizes a dynamic dictionary, facilitated by a queue and a momentum-updated encoder. Its distinguishing innovation lies in the sustenance of a coherent and evolving representation domain, enhancing the efficacy of contrastive learning. Such a strategy augments the quality of acquired representations, proving especially valuable in environments with a limited labeled dataset.

SimCLR, presented by Chen et al. [16], champions a relatively lucid yet potent framework. By employing data augmentation as a mode of self-supervision, SimCLR endeavors to maximize the affinity between variedly augmented interpretations of the same data instance within the latent domain. By obviating the need for intricate architectures or memory repositories, it presents itself as a more streamlined and scalable solution relative to its self-supervised counterparts.

Both MoCo and SimCLR have epitomized the potential of self-supervised learning, at times surpassing supervised methodologies in specific arenas. Their salient contributions underscore the feasibility of leveraging unlabeled datasets to generate representations that serve a multitude of downstream applications, substantially mitigating the onus of data annotation.

In spite of the evident promise exhibited by self-supervised learning modalities, there is a conspicuous dearth of scholarly contributions in the realm of energy consumption forecasting, to the best of our current understanding. A plausible impediment could be the intricate nature of pinpointing suitable pseudo-tasks for guiding the model’s preliminary training. Concurrently, there exists the challenge of architecting an apt network structure that adeptly amalgamates facets of both self-supervised and supervised learning paradigms.

In recent years, the advent of the transformer architecture [17] has catalyzed a renaissance in deep learning research. Owing to its remarkable feature extraction prowess, the architecture has found prolific applications in domains ranging from machine translation [18] to image processing [19,20]. A spate of studies has also explored its potential in time series analysis [21,22], corroborating its efficacy therein. Given these developments, it is reasonable to posit that transformer derivatives are apt candidates for integrative training spanning self-supervised and supervised learning paradigms.

Drawing upon the principles of self-supervised learning, we have architected a robust transformer-based framework termed as contrastive transformer networks (CTNs). This architecture embarks on an unsupervised pre-training trajectory, leveraging pseudo-tasks to attain optimally initialized network weights. Subsequently, a traditional supervised training regimen is employed for fine-tuning. Empirical evaluations underscore the potency of our proposed paradigm and the inherent feature extraction capabilities of CTNs. Notably, our architecture outperforms extant methodologies in forecasting future building energy consumption, thereby cementing its position as a promising avenue for advancements in building energy consumption prediction.

The contributions of this research can be summarized as follows:

1. We have designed efficient contrastive transformer networks (CTNs) for both self-supervised and supervised learning.
2. We have introduced self-supervised learning methods into the field of building energy consumption prediction, reducing the dependency of deep learning algorithms on the number of training data.
3. By combining the network architecture and self-supervised learning methods, we have designed an effective algorithm for predicting building energy consumption.

## 2. Related Works

### 2.1. Transformer

Transformer networks, initially proposed by Vaswani et al. [17], have revolutionized various fields of machine learning, including natural language processing and computer vision. The core concept behind the Transformer architecture is the self-attention mechanism, which enables the model to consider other parts of the input when processing a specific element.

The Transformer architecture consists of an encoder and a decoder. Both the encoder and the decoder are comprised of multiple identical layers, which utilize multi-head self-attention and feed-forward neural networks. The architecture employs positional encoding to infuse the sequence order into its representation.

Self-attention allows the network to focus on different parts of the input by computing a weighted sum of all input elements, guided by the attention scores. Formally, given a query *Q*, key *K*, and value *V* matrices, the attention is calculated as follows:

$$\mathrm{Attention}\left(Q,K,V\right)=\mathrm{Softmax}\left(\frac{QK^{⊤}}{\sqrt{d_{k}}}\right)V$$

where $d_{k}$ is the dimensionality of the key.

Transformers have been extensively applied in tasks such as machine translation [23], text summarization [24], and even in remote sensing for object detection [25]. Their parallelizable nature and ability to capture long-range dependencies have made them a popular choice for many sequence-to-sequence tasks.

Several variants and extensions of the Transformer architecture have been proposed to improve its efficiency and applicability, such as the BERT [26] for language understanding, and Vision Transformer (ViT) [27] for image classification tasks.

### 2.2. Temporal and Contextual Contrasting Method

Unsupervised representation learning for time-series data has been a challenge that researchers have been attempting to address for years. One significant contribution in this realm is the temporal and contextual contrasting (TCC) framework [28]. This method aims to learn robust representations from unlabeled time-series data.

The TCC framework initially processes raw time-series data into two different yet correlated views using both weak and strong augmentations. The first major innovation of this framework is the temporal contrasting module, designed to capture robust temporal dynamics. It does so by setting up a challenging cross-view prediction task. The framework then further refines these temporal representations using a contextual contrasting module, designed to maximize the similarity among different contexts within the same sample while minimizing similarities across different samples.

TCC has been applied to multiple real-world time-series datasets. The empirical results demonstrate that even a simple linear classifier, when trained on the features learned by TCC, can perform comparably to supervised methods. The framework has also shown promise in scenarios involving few-labeled data and transfer learning, thus proving its versatility and efficiency.

While other unsupervised learning methods focus mainly on either temporal dynamics or contextual information, TCC seamlessly integrates both. This dual focus allows for a more nuanced and robust feature representation, enabling the framework to outperform several existing methods in various applications.

## 3. Methods

**Figure 1:**
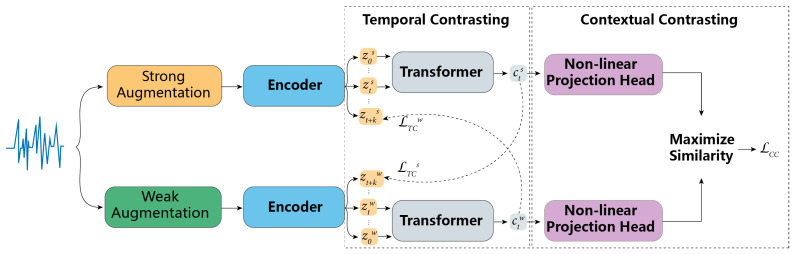
Structure of the proposed contrastive transformer networks (CTNs).

As illustrated in Figure 1, the contrastive transformer network (CTN) presents a sophisticated multi-stage architecture:

1. Data Augmentation: The input data undergo two distinct augmentation processes:
  - Strong Augmentation: Incorporates a permutation-and-jitter approach.
  - Weak Augmentation: Introduces random perturbations and amplifying data’s scale.
2. Encoding Phase: Both the strongly and weakly augmented data are processed through dedicated “Encoder” blocks, resulting in latent vectors z. Here, we adopt a transformer network instead of a convolutional network as the encoder to better capture the series information of data.
3. Temporal Contrasting: Latent vectors are passed through a “Transformer” block to capture temporal dependencies, producing embeddings c. c is restricted by the temporal contrasting loss $L_{TC}$.
4. Contextual Contrasting: Embeddings undergo a “Non-linear Projection Head” to project them into a space where similarity is maximized, leading to the final loss $L_{CC}$.

The CTN framework leverages both temporal and contextual contrasting to yield rich representations, making the network adept for subsequent downstream tasks.

### 3.1. Data Augmentation and Encoding

In the domain of contrastive learning, an essential innovation is the dual data augmentation scheme proposed in the TCC [28]. The TCC methodology employs two distinct families of augmentations, $T_{w}$ and $T_{s}$, to generate weak and strong augmented views, $x_{w}$ and $x_{s}$, of each sample *x*. By contrasting these two distinct perspectives, the model is enabled to learn more robust features.

For the weak augmentation, we manipulate the input signal by introducing random perturbations and amplifying its scale. In contrast, the strong augmentation strategy incorporates a permutation-and-jitter approach. This involves segmenting the signal into a randomly determined number of partitions, capped at *M*, followed by their random rearrangement. Subsequently, random fluctuations are added to the rearranged signal. It is crucial to tailor the augmentation parameters in accordance with the specific characteristics of the time-series dataset. For instance, when segmenting the signal, the upper limit *M* should be adjusted based on the sequence length and higher values of *M* are preferable for longer sequences. Similarly, the magnitude of the jitter should be considerably lower for normalized datasets as compared to unnormalized ones.

While the data augmentation approach contributes to the effectiveness of the contrastive model, it is important to clarify that this aspect of our research adopts TCC’s methodology and is not an original contribution. Contrary to TCC, we diverge by implementing a Transformer-based encoder instead of the conventional three-block convolutional structure. CTN opts for the Transformer architecture to mitigate potential information loss and to more effectively focus on individual features within each sample. Mathematically, the Transformer encoder maps an input *x* into a high-dimensional latent space *z* via a function $f_{\mathrm{enc}}$, such that

(1) $$z=f_{\mathrm{enc}}\left(x\right)$$

This modification aims to address limitations in convolutional networks, particularly when dealing with inputs of shorter signal lengths. Through the integration of the Transformer encoder, our methodology aspires to enhance both the robustness and adaptability of the learned representations for various downstream tasks.

We represent the high-dimensional latent space as $z=[z_{1},z_{2},\ldots ,z_{T}]$, with *T* denoting the total number of timesteps and each $z_{i}$ being a *d*-dimensional feature vector. From this representation, we obtain $z^{s}$ and $z^{w}$ corresponding to the strong and weak augmented views, respectively. These are subsequently input into the temporal contrasting module for further analysis.

### 3.2. Temporal Contrasting

The Temporal Contrasting module utilizes a contrastive loss function in combination with an autoregressive model to capture temporal dynamics in the latent feature space. Given a set of latent vectors *z*, the autoregressive model, denoted as $f_{\mathrm{ar}}$, aggregates all instances of *z* up to time *t* to produce a context vector $c_{t}=f_{\mathrm{ar}}\left(z\leq t\right)$. This vector resides in an *h*-dimensional hidden space, i.e., $c_{t}\in R^{h}$. Subsequently, this context vector is employed to forecast the latent states from $z_{t+1}$ to $z_{t+k}$, where 1<k≤K. For such predictive modeling, we apply a log-bilinear function defined as $f_{k}\left(x_{t+k},c_{t}\right)=\exp\left({\left(W_{k}\left(c_{t}\right)\right)}^{T}z_{t+k}\right)$, where $W_{k}$ is a linear transformation that maps $c_{t}$ back to the original latent space, or $W_{k}:R^{h}\to R^{d}$.

In our methodology, strong augmentation yields context vectors $c_{t}^{s}$ while weak augmentation provides $c_{t}^{w}$. We introduce a challenging cross-view prediction task that employs $c_{t}^{s}$ from the strong augmentation to anticipate future latent states in the weak augmented sequence $z_{t+k}^{w}$, and vice versa. The contrastive loss aims to minimize the cosine similarity between the predicted and true latent vectors of the same sample, while maximizing the similarity with alternative samples $N_{t,k}$ in the minibatch. We accordingly derive the loss terms $L_{TC}^{s}$ and $L_{TC}^{w}$ as Equations (2) and (3) show them.

Equation (2) represents the loss incurred when the context vector derived from the strong augmentation, denoted as $c_{t}^{s}$, is utilized to predict future latent states in the weak augmented sequence, symbolized by $z_{t+k}^{w}$. The term inside the exponential function, ${\left(W_{k}\left(c_{t}^{s}\right)\right)}^{T}z_{t+k}^{w}$, calculates the dot product between the transformed context vector and the future weak latent state. The objective here is to maximize the similarity of the dot product with the actual future state while reducing its similarity with other alternative samples, $N_{t,k}$, present in the minibatch.

(2) $$L_{TC}^{s}=-\frac{1}{K}\sum_{k=1}^{K}\log\frac{\exp\left({\left(W_{k}\left(c_{t}^{s}\right)\right)}^{T}z_{t+k}^{w}\right)}{\sum_{n\in N_{t,k}}\exp\left({\left(W_{k}\left(c_{t}^{s}\right)\right)}^{T}z_{n}^{w}\right)}$$

Conversely, Equation (3) elucidates the loss when the weak augmentation’s context vector, $c_{t}^{w}$, is employed to predict future latent states in the strong augmented sequence, denoted by $z_{t+k}^{s}$. Similarly, the term inside the exponential function, ${\left(W_{k}\left(c_{t}^{w}\right)\right)}^{T}z_{t+k}^{s}$, signifies the dot product between the weak context vector and the future strong latent state. The objective remains consistent: enhancing the similarity of the dot product with the correct future state and diminishing its similarity with the alternative samples, $N_{t,k}$, in the minibatch.

(3) $$L_{TC}^{w}=-\frac{1}{K}\sum_{k=1}^{K}\log\frac{\exp\left({\left(W_{k}\left(c_{t}^{w}\right)\right)}^{T}z_{t+k}^{s}\right)}{\sum_{n\in N_{t,k}}\exp\left({\left(W_{k}\left(c_{t}^{w}\right)\right)}^{T}z_{n}^{s}\right)}$$

Similar to the encoder architecture, we continue to employ a Transformer model to encode the latent vectors *z*. The resulting context vectors $c_{t}^{s}$ and $c_{t}^{w}$ are subsequently fed into the ensuing contextual contrasting module for further processing.

### 3.3. Contextual Contrasting

We extend our methodology by introducing a Contextual Contrasting module designed to yield more discriminative feature representations. Initially, we employ a non-linear projection head, similar to the approach in Chen et al.’s work [16], to map the context vectors into the contrasting space.

The concept of using a non-linear projection head is critical as it allows for a more robust transformation of the original context vectors. By doing so, we can better harness the discriminative information contained within the vectors, positioning them optimally in the contrasting space. This methodology is inspired by the success seen in the work by Chen et al. [16], where such projections have shown significant benefits in the realm of self-supervised learning.

For a batch comprising *N* samples, each having two augmented views, we obtain 2N context vectors. Let $c_{t}^{i}$ represent a specific context, and $c_{t}^{i+}$ denote its positive counterpart, generated from the other augmented view of the same sample. Thus, $\left(c_{t}^{i},c_{t}^{i+}\right)$ constitutes a positive pair, while the remaining (2N−2) contexts from different inputs form negative pairs.

In simpler terms, for every sample in our batch, we generate two context vectors from two augmented views. A positive pair is formed when these two context vectors are derived from the same sample. On the other hand, any context vector, when paired with another vector from a different sample, forms a negative pair. This approach ensures a balanced representation of both similarity (positive pairs) and dissimilarity (negative pairs) within our contrasting space.

To leverage this configuration, we define a Contextual Contrasting loss function, denoted as $L_{CC}$ as Equation (4) shows.

(4) $$L_{CC}=-\sum_{i=1}^{N}\log\frac{\exp\left(\mathrm{sim}\left(c_{t}^{i},c_{t}^{i^{+}}\right)/\tau \right)}{\sum_{m=1}^{2N}I_{\left[m\neq i\right]}\exp\left(\mathrm{sim}\left(c_{t}^{i},c_{t}^{m}\right)/\tau \right)}$$

The above equation is the heart of our contrastive framework. It quantifies the disparity between the similarity score of the positive pairs and that of the negative pairs. The objective is to make sure that positive pairs have high similarity scores compared to any negative pairs. The function sim computes the similarity, and the term τ serves as a temperature parameter, providing a scaling factor to the similarity values.

The similarity is computed using a normalized dot product, given by

(5) $$\mathrm{sim}\left(u,v\right)=\frac{u^{T}v}{∥u∥∥v∥}$$

This is a fairly standard way to measure similarity in high-dimensional spaces. By normalizing both vectors and then computing their dot product, we ensure that the similarity score remains in a bounded range and provides a clear measure of how alike two vectors are.

An indicator function 1[m≠i]∈{0,1} is utilized, which equals 1 when m≠i. Additionally, we introduce a temperature parameter τ to modulate the loss.

The indicator function is a simple way to exclude the main diagonal elements (which are the self-similarities of the vectors) from the computation, ensuring that we do not compare a vector with itself. The temperature parameter τ is pivotal in controlling the sharpness of the probability distribution, thereby influencing the convergence and performance of the contrastive task.

As Equation (6) shows, the overall self-supervised learning objective consists of the sum of the Temporal Contrasting losses and the Contextual Contrasting loss, weighted by scalar hyperparameters $\lambda_{1}$ and $\lambda_{2}$ that indicate the importance of each respective loss component.

(6) $$L=\lambda_{1}\cdot \left(L_{TC}^{s}+L_{TC}^{w}\right)+\lambda_{2}\cdot L_{CC}$$

The final learning objective, as described above, beautifully brings together the individual contrasting loss components. The scalar hyperparameters allow for a nuanced control over the contribution of each loss component, enabling the model to be tailored according to specific requirements or based on empirical evaluations.

Through the integration of these modules, the contrastive transformer network (CTN) is proficient at leveraging the initial samples for feature extraction, thereby facilitating the training of downstream tasks.

## 4. Experimental Setup

### 4.1. Dataset

For our empirical analysis, we procured a comprehensive dataset detailing the electricity consumption of the Hangzhou International Expo Center (HIEC) in China. Spread over a colossal area of 850,000 square meters, the HIEC, by the conclusion of December 2022, has been the venue for an impressive tally of over 7400 conferences and 260 exhibitions. Given its significant energy requirements, the HIEC stands as a prime candidate for in-depth power consumption prognostication studies.

The dataset chronicles the daily electricity demand of the HIEC spanning from 1 September 2019 to 30 December 2022. The infrastructural layout of the HIEC bifurcates it into three disparate sectors: conference, hotel, and exhibition domains. These sectors, by virtue of their unique operational purposes, are naturally expected to exhibit divergent electricity consumption trajectories. However, a compelling congruence was discerned in the power uptake patterns of air conditioning systems across these sectors. Consequently, our dataset meticulously encompasses the air conditioning consumption metrics from each of these domains.

To ascertain the integrity and precision of our recorded data, we harnessed sophisticated remote sensing methodologies. This entailed deploying an ensemble of cutting-edge sensors, judiciously positioned across the establishment. The instrumentation—a synergistic blend of piezoelectric energy meters and infrared thermal sensors—empowered us to chronicle instantaneous power consumption with impeccable accuracy. Specifically, the piezoelectric meters adeptly registered the nuances in electrical demand, while the infrared thermal apparatuses oversaw the performance and power metrics of the HVAC (Heating, Ventilation, and Air Conditioning) systems, with a focus on air conditioning units.

Aware of the significant impact of climatic conditions on energy consumption, especially in HVAC systems, our dataset was complemented with historical weather data. This information was sourced from the China Meteorological Data Service Center, providing detailed records of daily maximum and minimum temperatures. By integrating these weather variables, we not only enhanced the depth of our dataset but also underscored the nuanced relationship between temperature variations and the energy requirements of air conditioning units.

A sample display of the dataset can be found in Table 1. Different areas were categorically encoded, i.e., 0 means conference area, 1 means hotel area, and 2 means exhibition area. In the dataset, every record can be identified by the “Date” and “Area” columns. The “Consumption(kWh)” column is the consumption of air conditioners, which is the target value to forecast. The “Max_temperature(°C)” and “Min_temperature(°C)” columns are auxiliary input variables to help forecasting.

**Figure 2:**
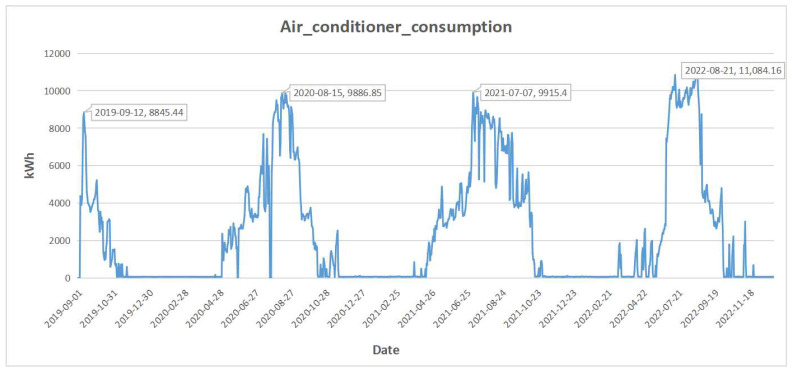
Daily consumption of the air conditioners in hotel area.

We also show part of the dataset in Figure 2. In the hotel area, the consumption of air conditioners is visibly higher than other months in summer, which indicates that using months as part of the input features can help the prediction. This pattern is similar in two other areas. And it implies that temperature is an important variable that influences the power consumption of air conditioners. Also, some anomaly points (zero values) in the dataset are shown in Figure 2, so we cleaned the data before building the training and testing set. For those points with zero values, we replaced them with the mean of the normal points neighboring the anomaly points. And the neighboring window size is set to 14.

### 4.2. Comparative Methods

To validate the utility of proposed CTN model, we used five baseline methods for comparison in the experiments. First, we adopted a simple model that uses the last known target value to make a prediction, named as Baseline. Then, we adopted a designed LSTM network [29] and a designed gated recurrent unit (GRU) neural network [30] for comparison. Also, we used the classical self-supervised method SimCLR for comparison. LSTM and GRU are both types of recurrent neural networks (RNNs) that have been specifically designed to address the problem of vanishing gradients in traditional RNNs. Both GRU and LSTM employ gating mechanisms that enable them to selectively retain or discard information from previous time steps. Such mechanisms facilitate the preservation of long-term dependencies in time series data. While GRU excels in capturing these dependencies, it has occasionally been outperformed by LSTM in certain scenarios. Therefore, utilizing GRU and LSTM offers a reflection of the performance of classic deep learning prediction methods on this task. It is worth noting that we implemented time-series specific augmentations to adapt SimCLR to our application as it was originally designed for images.

To further demonstrate the efficacy of the CTN model, we introduce two other baseline methods for comparison: Random and Supervised. The Random approach initializes all layers within the CTN model with random weights and subsequently freezes them; only the final non-linear layer undergoes updates during training. Conversely, the Supervised approach bypasses any pre-training of the CTN and trains it directly with labeled data. Comparing the CTN’s performance against these methods provides clear evidence of the benefits derived from its self-supervised learning phase in enhancing prediction accuracy.

We posit that employing these six comparative methods sufficiently underscores the architectural merits of CTN as well as its advantages in self-supervised learning.

### 4.3. Implementation Details

To capture the intricate relationship between time and electricity consumption, we undertook specific preprocessing steps during dataset construction. Initially, the “Date” attribute was transformed into a “Month” variable. Subsequently, this “Month” variable was combined with other features, namely “consumption”, “Max temperature”, and “Min temperature”, to structure the dataset into four-dimensional vectors.

A sliding window of length 7 was established to capture sequential temporal dependencies, where data within each window served as historical context and the “consumption” value of the subsequent day was treated as the prediction target. By employing this scheme, the dataset was segmented into multiple overlapping samples.

Out of our dataset, a total number of 3515 samples were extracted. Every sample corresponds to 7 days of data, capturing four distinct features for each day. Mathematically speaking, our dataset is a tensor whose shape is (3515,7,4).

For enhanced model training and evaluation, the data samples underwent a random split in a 4:1 ratio, designating the latter fraction for the testing set. The larger portion was subsequently subdivided into training and validation sets at a 3:1 distribution.

Following our data split method, we obtained a distribution of 2109 samples for the training set, 703 samples for the validation set, and 703 samples for the testing set.

All experiments were implemented on a personal computer with 32 GB RAM, and an RTX 3090ti GPU manufactured by NVIDIA Corporation, headquartered in Santa Clara, CA, USA. The coding environment wasPytorch [31]. We repeated all the experiments over five times and recorded the average results.

The evaluation metrics are root mean squared error (RMSE) and mean absolute percentage error (MAPE). RMSE is a measure of the average deviation of the predicted values from the actual values. As shown in Equation (7), RMSE is computed by three variables, $y_{i}$ is the actual value of the i-th observation, $\hat{y_{i}}$ is the predicted value of the i-th observation, and *n* is the total number of observations.

(7) $$\mathrm{RMSE}=\sqrt{\frac{1}{n}\sum_{i=1}^{n}{\left(y_{i}-{\hat{y}}_{i}\right)}^{2}}$$

MAPE is a measure of the percentage difference between the predicted and actual values. Equation (8) shows how MAPE is computed, $y_{i}$ is the actual value of the i-th observation, $\hat{y_{i}}$ is the predicted value of the i-th observation, and *n* is the total number of observations:

(8) $$\mathrm{MAPE}=\frac{100\%}{n}\sum_{i=1}^{n}\left|\frac{y_{i}-{\hat{y}}_{i}}{y_{i}}\right|$$

## 5. Results

The experimental results are presented in Table 2, which documents the RMSE and MAPE metrics for both the comparative methods and CTN in the prediction task.

From an RMSE perspective, the CTN demonstrates superior performance with the lowest error of 2117.31, slightly surpassing the Supervised method with an RMSE of 2156.47. This suggests that our self-supervised learning enhances model proficiency, particularly in limited data contexts. LSTM and GRU, while respectable, do not exceed the CTN’s performance. The Random method’s high RMSE of 2919.22 highlights its inefficacy and reaffirms the value of our pre-training strategy.

In terms of MAPE, the Supervised method slightly outperforms with a score of 0.74, but the CTN closely follows at 0.75, reiterating its robust predictive power. The Random method’s high MAPE of 3.68 further elucidates its predictive shortcomings.

The results underscore the CTN framework’s potential, outstripping traditional models like LSTM and GRU. SimCLR, another self-supervised approach, posts an RMSE of 2263.72 and MAPE of 0.96. While it surpasses models like LSTM and GRU, it does not match CTN’s prowess. The disparity suggests the limitations of relying solely on contrastive learning, like SimCLR, without the CTN’s integrated advantages.

The uniqueness of the CTN lies in its fusion of contrastive self-supervised learning and transformers, enhancing its ability to discern intricate temporal patterns crucial for forecasting tasks like power consumption. SimCLR emphasizes instance discrimination without accentuating temporal correlations, possibly explaining its marginally elevated error metrics. Moreover, the CTN’s Contextual Contrasting module refines its feature representations, strengthening its overall performance. This consolidative approach ensures that the CTN achieves a comprehensive contrasting technique, refining feature robustness.

Conclusively, while SimCLR is an effective self-supervised method, electricity consumption forecasting appears to benefit more from CTN’s comprehensive design. This emphasizes the importance of tailoring self-supervised approaches to specific prediction challenges.

## 6. Conclusions

In this study, we developed the contrastive transformer network (CTN) for predicting energy consumption in large buildings using small sample data. The model leverages an efficient feature extraction architecture and self-supervised learning to improve predictive accuracy. This research is important because it presents a new approach to tackle the problem of small sample energy prediction, offering a valuable alternative when large-scale data are unavailable or expensive to acquire. Our empirical results demonstrate that the CTN is a superior method in this domain, especially in scenarios wherein data are limited. For our small dataset, the CTN obtained 2117.31 in terms of RMSE, outperforming other baseline methods. Going forward, we plan to explore more advanced self-supervised techniques and alternative architectures to further refine the model’s predictive capabilities.

## Figures and Tables

**Table 1:** Daily samples of the dataset used in the paper.

Date	Consumption (kWh)	Max_Temperature (°C)	Min_Temperature (°C)	Area
5 January 2022	59.39	11	6	1
16 November 2021	667.93	19	10	2
4 May 2020	2366.46	36	20	1
15 November 2021	584.63	19	9	2
3 October 2021	5894.18	34	21	0

**Table 2:** Results of the comparative methods. The best results are shown in bold.

Method	Baseline	LSTM	GRU	SimCLR	Random	Supervised	CTN
RMSE (kWh)	2536.55	2370.40	2416.31	2263.72	2919.22	2156.47	**2117.31**
MAPE	1.08	0.98	1.06	0.96	3.68	**0.74**	0.75

## Data Availability

Restrictions apply to the availability of these data. Data was obtained from Zhejiang Dongguan Information Technology Co., Ltd. and are available from the authors with the permission of Zhejiang Dongguan Information Technology Co., Ltd.

## Abbreviations

The following abbreviations are used in this manuscript:

RNNs	Recurrent Neural Networks
S2S	Sequence-to-Sequence
LSTM	Long Short-Term Memory
KF	Kalman Filter
SVM	Support Vector Machine
GRU	Gated Recurrent Unit
CTN	Contrastive Transformer Network
MoCo	Momentum Contrast
SimCLR	Simple Contrastive Learning of Visual Representations
